# Towards Next-Generation Sequencing as a First-Tier Diagnostic Test for Fructose-1,6-Bisphosphatase Deficiency

**DOI:** 10.3390/metabo16010056

**Published:** 2026-01-08

**Authors:** Nadine Yazbeck, Abir Barhoumi, Pascale E. Karam

**Affiliations:** 1 Division of Pediatric Gastroenterology and Nutrition, Department of Pediatrics and Adolescent Medicine, American University of Beirut Medical Center, Beirut P.O. Box 2020, Lebanon; ny12@aub.edu.lb; 2 Department of Nutrition, American University of Beirut Medical Center, Beirut P.O. Box 2020, Lebanon; an04@aub.edu.lb; 3 Inherited Metabolic Diseases Program, Department of Pediatrics and Adolescent Medicine, American University of Beirut Medical Center, Beirut P.O. Box 2020, Lebanon

**Keywords:** next generation sequencing, diagnostics, fructose-1,6-bisphosphatase, *FBP1*, inborn errors of metabolism

## Abstract

**Background:** Advances in genomic technologies combined with tandem mass newborn screening have enabled early detection and management of several common inborn errors of metabolism. Fructose-1,6-bisphosphatase deficiency, an autosomal recessive treatable disorder reported in around 150 patients worldwide, remains underdiagnosed despite an excellent prognosis with early detection. Although common in highly consanguineous populations, diagnosis is often delayed due to the non-specific clinical and biochemical profile. **Methods:** This report explores the diagnostic pathway using first-tier next-generation sequencing of three novel cases of fructose-1,6-bisphosphatase deficiency in a tertiary care center in Lebanon. **Results:** Two patients were diagnosed with first-tier exome sequencing within one month of presentation and had an excellent outcome at 6 years of follow-up. The third patient, undiagnosed for 10 years, suffered from neurological sequalae. The molecular profile was remarkable in two patients for exon 2 deletion in the *FBP1* gene, a founder mutation reported in Turkish and Armenian patients, and a rare frameshift mutation in the third case. **Conclusions:** The use of next-generation sequencing as as a first-tier test for FBP deficiency is a non-invasive and rapid method for early diagnosis and management of this rare yet treatable disorder. It can detect both disease-causing variants and large deletions, founder mutations as well, delineating the molecular profile in populations where this disorder is highly prevalent.

## 1. Introduction

**Figure 1:**
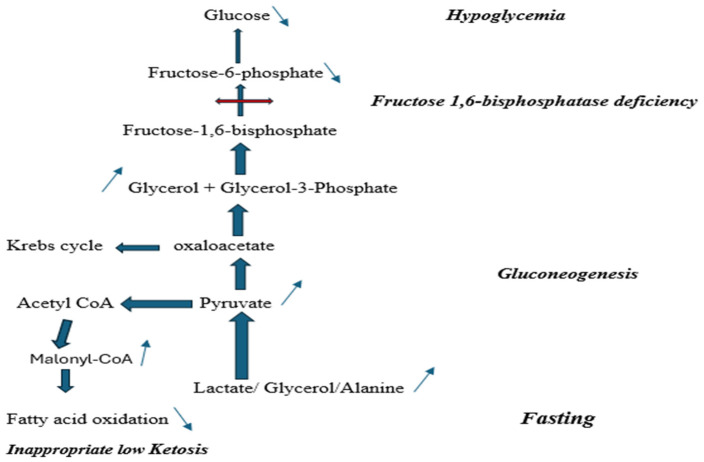
Fructose-1,6-bisphosphatase deficiency biochemical pathway.

The screening and diagnostic pathways for inborn errors of metabolism have been radically transformed in recent years by the emergence of advanced genomic technologies. Traditionally, diagnosis relied on biochemical investigations, enzyme assays in cultured cells or tissues, and a strong index of suspicion by the clinician. In the early 1990s, the introduction of newborn screening using tandem mass spectrometry (MS/MS) enabled early detection and management of common inborn errors of metabolism, such as classic phenylketonuria, maple syrup urine disease, and fatty-acid oxidation defects, thereby significantly improving outcomes [1]. However, other treatable disorders with excellent prognosis remain underdiagnosed. Within this broad context, the gluconeogenic defect of fructose-1,6- bisphosphatase (FBP) deficiency warrants particular attention. This is an extremely rare autosomal recessive disorder, caused by biallelic pathogenic variants in the *FBP1* gene, affecting the last step of gluconeogenesis (Figure 1); thus, impairing glucose production from non-carbohydrate sources associated with fasting hypoglycemia and accumulation of lactate, glycerol, glycerol-3-phosphate, and ketones [2]. Approximately 150 affected individuals are described in the literature to date. Estimated frequencies range from ~1:350,000 in the Netherlands to <1:900,000 in France [3] while prevalence is reported as low as ~1:1,310,034 in the Chinese population [4].

Although considered rare worldwide [4], this disorder is frequently encountered in Middle Eastern and North African countries with high consanguinity rates [5], such as Turkey [6], Saudi Arabia [7], and Morocco [8]. In Lebanon, consanguineous marriages reach 35.5% of the population [9], contributing to a higher prevalence of autosomal recessive disorders.

Clinical presentation is characterized by hepatomegaly, recurrent episodes of hypoglycemia upon fasting, lactic acidosis, ketosis, and hyperalaninemia. Added to that, acute crises are often precipitated by fasting or fever, and many children experience recurrent episodes before diagnosis. If unrecognized, it can lead to potentially serious neurologic sequelae.

Several recent cohort studies document significant delays between symptom onset and definitive diagnosis. This is due both to the rarity of this disorder and the fact that its clinical features can mimic more common conditions such as glycogen storage diseases, mitochondrial disorders, or fatty acid oxidation defects [3]. 

Up to the last decade, diagnosis of FBP deficiency was established by enzyme activity assays in leucocytes or liver tissue, which are invasive and not widely available [10]. In contrast, in the current era, the availability of next-generation sequencing (NGS) enables rapid, non-invasive identification of pathogenic *FBP1* variants even in patients with atypical biochemical profiles. Evidence from recent studies demonstrates that early genomic testing can shorten diagnostic delays, improve accuracy, and guide timely interventions [11,12,13]. Given the heterogeneity and non-specificity of biochemical findings, and the potential for serious complications if untreated, FBP deficiency could be an ideal candidate for first-tier genomic testing in high-risk populations [14].

Here, we describe the diagnostic pathway and outcome of three novel cases of FBP deficiency identified through NGS in a tertiary care center in Lebanon. This study explores the implications for diagnostic workflows and precision screening for FBP deficiency in populations characterized by high consanguinity.

## 2. Materials and Methods

A retrospective review of charts of all patients with a confirmed diagnosis of FBP deficiency that were followed at the Inherited Metabolic Diseases Program, Department of Pediatrics and Adolescent Medicine at the American University of Beirut Medical Center- Lebanon, between 2010 and 2023. Age of onset, clinical presentation, biochemical and molecular investigations were systematically recorded, along with age at diagnosis and subsequent outcome.

Molecular studies were performed at reference genetic laboratories. Since 2010, NGS has been available through a reference genetics laboratory in Lebanon or by outsourcing samples to Centogene in Germany.

The overall whole exome sequencing methodology with copy number variation used in this study follows standard clinical exome sequencing principles. The generated library is sequenced on an Illumina platform to obtain at least 20× coverage depth targeting > 98% of the coding RefSeq. At the reference genetic laboratory in Lebanon, reads were aligned to hg19 using BWA v0.6.1, followed by indel realignment, duplicate marking, and base quality recalibration using GATK v1.6, Picard v1.62, and SAMtools v0.1.18. Variants were called using GATK UnifiedGenotyper and annotated with Ensembl and dbSNP135. At Centogene, an in-house bioinformatics pipeline, including read alignment to GRCh37/hg19 genome assembly, variant calling, annotation, and comprehensive variant filtering, is applied. Variants are categorized according to ACMG/AMP guidelines in addition to ClinGen recommendations. The copy number variation detection software used has a sensitivity of more than 95%. Variants with low sequencing quality and/or unclear zygosity are confirmed by orthogonal methods.

## 3. Results (Table 1)

Patient 1 was first evaluated at our center at 13 months of age following an episode of hypoglycemia (35 mg/dL; Normal: 76–110 mg/dL), severe lactic acidosis (8 mmol/L; Normal: 0.6–2.2 mmol/L), and hypertriglyceridemia (740 mg/dL; Normal less than 150) that occurred after surgical reduction in an intestinal intussusception at another hospital, complicated by severe protracted vomiting diagnosed as suspected acute pancreatitis.

She was born to parents who are first-degree cousins. Her physical exam was normal. Basic metabolic investigations, including plasma amino acids chromatography, acylcarnitine profile, and urine organic acids, were unremarkable.

She was subsequently lost to follow-up for 3 years and 5 months. She presented again at 4 years and 6 months with a history of recurrent hypoglycemic episodes precipitated by infections and poor feeding. These crises were characterized by severe lactic acidosis and hypertriglyceridemia and resolved within 2 to 3 days with intravenous glucose infusion. On re-evaluation, she had moderate intellectual disability, speech difficulties, and hepatomegaly.

Fasting studies at 4 h for suspected glycogen storage disease type I were unremarkable, but after 12 h of fasting, the patient developed profound hypoglycemia (glucose 27 mg/dL; Normal: 76–110 mg/dL), marked metabolic acidosis (bicarbonate 8 mmol/L; Normal 24–30 mmol/L), and significantly elevated transaminases (AST 609 IU/L (Normal: 0–65 IU/L), ALT 355 IU/L (Normal: 0–50 IU/L]). Hepatomegaly was confirmed by ultrasound. Differential diagnoses initially included pyruvate carboxylase deficiency, mitochondrial defects, fatty acid oxidation defects, and glycogen storage diseases. Genetic testing was requested, but it was not performed for financial reasons. Meanwhile, a fructose-restricted diet and avoidance of fasting were recommended.

The diagnosis of FBP deficiency was finally confirmed at 10 years of age by NGS. Exome sequencing, performed at the reference genetic laboratory in Lebanon, revealed a homozygous deletion encompassing exon 2 of the *FBP1* gene (previously known as exon 1). Segregation analysis confirmed that both parents were heterozygous carriers of this deletion. At last follow-up, at 13 years of age, she continued to exhibit speech delay and moderate intellectual disability, without any further episodes of acute metabolic decompensation.

Patient 2 was a 2-year-old girl, born to consanguineous parents, who presented to the emergency department with fever, vomiting, and somnolence. One week prior to presentation, she was investigated at another hospital for a similar episode of fever and vomiting followed by loss of consciousness at home.

She was found to have hypoglycemia, metabolic acidosis, lactic acidosis, hyperuricemia, and hypertriglyceridemia. She received intravenous dextrose (10%) and bicarbonate infusions before being transferred to our center for further management. Upon physical exam, she was awake but irritable and was noted to have hepatomegaly. Her psychomotor development and growth parameters were appropriate for age.

Laboratory evaluation during dextrose infusion revealed a glucose level of 82 mg/dL (Normal: 76–110 mg/dL), uric acid 11.5 mg/dL (Normal: 2.5–6.6 mg/dL), triglycerides 862 mg/dL (less than 150 mg/dL), and arterial lactate 8.17 mmol/L (Normal: 0.6–2.2 mmol/L); liver transaminases were normal. Abdominal CT Scan demonstrated hepatomegaly with mild steatosis and no splenomegaly. The initial differential diagnosis included glycogen storage disease type I.

Exome sequencing conducted at the reference genetic laboratory in Lebanon, as a first-tier test, revealed a homozygous variant in the *FBP1* gene NM_000507.3: c.807delG (p.Lys270Argfs*7), confirming the diagnosis of FBP deficiency. Parental genotyping confirmed heterozygosity for the same deletion in both parents. Parents were educated about avoiding fasting, fructose, sucrose, and sorbitol, in addition to dietary precautions during acute illnesses. At her last follow-up visit, at 6 years of age, she had normal growth and developmental milestones.

Patient 3 was an 8-month-old boy, born full term to non-consanguineous parents from the same village. The first hypoglycemic episode occurred at 4 months of age during acute gastroenteritis. It was associated with metabolic acidosis and ketosis that were thought to be related to dehydration and poor feeding. He subsequently experienced two additional hypoglycemic episodes with lactic acidosis and hypertriglyceridemia, after which he was referred to our center. He had hepatomegaly on physical exam with normal growth and development.

Outside of acute episodes, baseline metabolic investigations, at 4 h of fasting for suspected glycogen storage disease type I, including blood glucose, lactate, and alanine, were normal. At this point, the differential diagnosis was broad, and the parents were unable to afford additional testing.

Exome sequencing was performed as a first-line diagnostic test. Molecular analysis performed at Centogene laboratory in Germany revealed a homozygous deletion of exon 2 in the *FBP1* gene (previously known as exon 1), confirming the diagnosis of FBP deficiency. Familial segregation studies confirmed heterozygous carrier status for the deletion in each parent. Dietary management was initiated with no subsequent acute metabolic crises. At the most recent follow-up, at 7 years of age, he demonstrated normal growth and good academic performance.

**Table 1:** Clinical, biochemical, and molecular characteristics of three Lebanese patients with FBP deficiency.

FBP Patients		Patient 1	Patient 2	Patient 3
**Age of onset**		13 months	2 years	4 months
**Age at Diagnosis**		10 years	2 years	8 months
**Time to Diagnosis**		9 years	1 month	4 months
**Presenting symptoms**		Lethargy episodes	Fever, vomiting, somnolence	Lethargy episode
**Biochemical findings (blood)**	Glucose (mg/dL) (Normal: 76–110)	27	40	32
	Lactic acid (mmol/L) (N: 0.6–2.2)	7	8.17	8.5
	Bicarbonate (mmol/L) (Normal: 24–30)	8	NA	5
	Triglycerides (mg/dL) (Normal: less than 150)	320	862	400
	Uric acid (mg/dL) (Normal: 2.5–6.6)	9	11.5	10
	AST/ALT (IU/L) (Normal: 0–65/0–50)	609/355	60/45	52/37
**Molecular findings**	Exome sequencing (NGS—Copy Number Variation)	Homozygous exon-1 deletion	Homozygous c.807delG	Homozygous exon-1 deletion
**Outcome at last follow-up**		At 10 years of age: moderate intellectual disability	At 6 years of age: normal growth, developmental milestones	At 7 years of age: normal growth, developmental milestones

## 4. Discussion

Several recent studies proposed NGS as a first-tier diagnostic modality for inborn errors of metabolism, either alone [15], in combination with metabolomics [16], or integrated with tandem mass spectrometry newborn screening [17]. In a Mexican cohort of patients with inborn errors of intermediary metabolism, whole exome sequencing by NGS achieved a diagnostic yield of 72.6% [18]. In parallel, in a study from our tertiary care center in Lebanon, 126 of 211 patients suspected of having an inborn error of metabolism were evaluated by NGS, with an overall diagnostic yield of 64.3% [19]. Diagnostic success varied according to disease category and testing strategy: targeted single-gene sequencing reached 75% yield in disorders with well-defined biochemical signatures, such as aminoacidopathies and organic acidemias, whereas whole-exome sequencing was particularly useful for complex cases, such as mitochondrial disorders.

FBP deficiency is an underdiagnosed yet treatable disorder, for which early diagnosis and management are key to a good outcome. Moreover, fewer than 200 patients have been reported worldwide; the disorder appears more common in populations with a high prevalence of consanguinity, such as Saudi Arabia and Turkey [6,7]. Our three new Lebanese cases expand the phenotypic and genotypic spectrum of FBP deficiency and illustrate the benefits of early NGS in reducing diagnostic lag.

Prior to the widespread use of NGS or directed gene panels, the diagnosis of FBP deficiency was often delayed. Lebigot et al. reported a mean age of diagnosis of three years, often after glycogen storage disorders and mitochondrial diseases were initially retained [10]. In a Saudi series, Salih et al. described a mean delay of 39.4 months in their study of 7 patients who were eventually diagnosed by NGS [20]. Recently, Ni et al. [4] highlighted the ability of whole exome sequencing to expand the mutation spectrum and facilitate genotype-phenotype correlation, which is critical for predicting clinical severity and guiding management.

**Figure 2:**
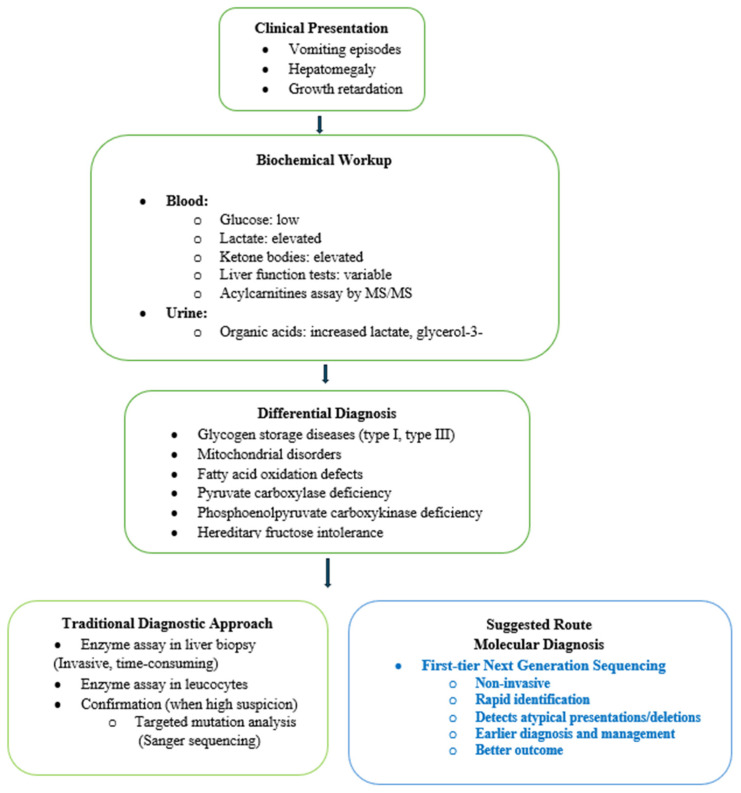
Diagnostic workflow for Fructose-1,6-bisphosphatase deficiency.

These delays reflect the nonspecific presentation of FBP deficiency and the need for extensive biochemical testing and stepwise gene-by-gene sequencing in the traditional diagnostic pathway. In populations with a high prevalence of FBP deficiency, performing exome sequencing by NGS, with integrated copy number variations, as a first-tier test (Figure 2) can provide a rapid and comprehensive method for diagnostic confirmation.

In our cohort, wo patients carried a homozygous exon 2 deletion (previously known as exon 1), which is a common pathogenic variant in Turkish patients and considered a founder mutation in Turkish and Armenian populations [5,6]. The third patient exhibited a rare homozygous variant, NM_000507.3: c.807delG (p.Lys270Argfs*7), reported once in the literature in a patient of unknown origin [5,21]. This variant results in a frameshift and premature truncation of the *FBP1* protein, consistent with a loss-of-function mechanism, which is the established cause of fructose-1,6-bisphosphatase deficiency [22,23]. Frameshift and truncating variants in *FBP1* are documented as pathogenic in ClinVar (e.g., c.779del; p.Gly260fs) and in clinical reports [5,21,24]. In addition, this specific Human Genome Variation Society variant has not been cataloged with a dedicated ClinVar accession; it is explicitly listed among disease-causing *FBP1* mutations in the original mutation spectrum reported by Herzog et al. [21]. It is subsequently curated and classified as a pathogenic frameshift variant in the comprehensive molecular review by Santer et al. [5], which summarizes confirmed *FBP1* deficiency–associated alleles from affected individuals. This variant is absent or extremely rare in population databases, consistent with rare autosomal recessive metabolic disorders [25]. Applying ACMG/AMP criteria therefore yields PVS1 (predicted null variant in a gene where loss-of-function is a known disease mechanism) and PM2 (absence from population databases), supporting a likely pathogenic classification [26,27].

The coexistence of both large deletion and frameshift variants within a single cohort reflects the genetic heterogeneity of FBP deficiency, even in a small country with high consanguinity rates. Recently, the literature increasingly supports the integration of genomic approaches into clinical workflows and newborn screening programs. Stark and Scott highlight the potential of genomic newborn screening to identify rare treatable conditions early in life [11]. Systematic reviews and large cohort studies have shown that whole exome and whole genome sequencing outperform traditional targeted methods in metabolic disorders and significantly influence clinical decision-making [12,13]. The clinical outcomes in our patients also highlight the utility of early use of NGS; two were diagnosed within one month of presentation, while the third experienced a longer diagnostic odyssey. This contrast underscores how first-tier genomic testing can enable timely intervention and prevent potentially irreversible complications.

We acknowledge a number of limitations inherent to this study. The small number of patients reflects the rarity of FBP deficiency and limits statistical analysis or generalizability. The retrospective design relies on available clinical and biochemical data, which may not have been collected uniformly and may introduce referral bias. Moreover, the identified *FBP1* variant NM_000507.3: c.807delG (p.Lys270Argfs*7) is supported by the existing literature and current classification guidelines; no functional validation was performed. Additionally, our findings may not be fully generalizable to other populations, as the study was conducted in a setting with high consanguinity, which may influence both disease prevalence and variant distribution. Finally, while the study supports the clinical utility of early NGS, no formal economic evaluation was performed to assess its cost-effectiveness as a first-tier diagnostic tool.

## 5. Conclusions

This report adds clinical and molecular evidence supporting the use of NGS with integrated copy-number analysis as a first-tier test for children with unexplained hepatomegaly, hypoglycemia, and metabolic ketoacidosis, to diagnose rare but treatable inborn errors of metabolism, such as FBP deficiency. This approach captures both pathogenic sequence variants and exon-level deletions, including founder mutations that may be enriched in populations with high rates of consanguinity. 

## Data Availability

All available data is published in the manuscript.

## Abbreviations

The following abbreviations are used in this manuscript:

MS/MS	tandem mass spectrometry
FBP	fructose-1,6-bisphosphatase
NGS	next-generation sequencing
